# Prediction models in multicenter studies: methodological aspects and current state of the art

**DOI:** 10.1186/2049-3258-73-S1-O3

**Published:** 2015-09-17

**Authors:** Laure Wynants, Sabine Van Huffel, Ben Van Calster

**Affiliations:** 1 KU Leuven, Leuven, Belgium

## 

Increasingly, multicenter datasets are being used to develop or evaluate clinical risk prediction models. Such models estimate an individual's probability that a certain disease or condition is present (diagnostic model) or that an event will occur in the future (prognostic model). Although multicenter studies enhance the generalizability of the model, the clustered nature of the data poses several methodological challenges. We will provide an up to date overview of good practices to overcome these challenges.

When determining the required sample size, the number of events per candidate variable (EPV) is crucial to prevent overfitting when building a prediction model. We extend the EPV guidelines to multicenter studies, acknowledging the clustered nature of the data. During data collection, measurements of variables may differ between centers due to various reasons, such as subjectivity of measurements, differences in equipment and differences in patient populations. We show how the residual intraclass correlation can be used to quantify the intercenter variability. When building a prediction model, the clustered nature of the data should be taken into account during the data analysis, e.g. by using mixed effect models and variables at the center level. Only mixed effect regression can result in a model that is simultaneously calibrated (i.e. gives accurate predicted probabilities) at the center level and the population level. We give the example of the ADNEX model that was built to distinguish between several types of adnexal masses. In the end, the performance of models may differ between centers. We present how to evaluate the predictive performance of models in clustered data and show extensions to existing techniques to evaluate discrimination, calibration and clinical utility, among others by the use of meta-analytic techniques.

